# The mite *Macrocheles robustulus* (Mesostigmata, Macrochelidae) a new promising natural enemy of *Haemonchus contortus* (Strongylida, Trichostrongylidae)

**DOI:** 10.1186/s13071-025-06990-x

**Published:** 2025-08-19

**Authors:** Adrien Bamière, Julie Petermann, Damien Morel, Philippe Jacquiet, Christelle Grisez

**Affiliations:** 1 UMR INRA/DGER 1225, Ecole Nationale Vétérinaire de Toulouse (ENVT), 23 Chemin des Capelles, 31076 Toulouse Cedex 03, France; 2 Bestico, 44860 Pont St Martin, France

**Keywords:** *Haemonchus contortus*, *Macrocheles robustulus*, Predatory behavior, Biological control, Pasture management

## Abstract

**Background:**

Antiparasitic resistance in sheep necessitates non-chemical strategies for gastrointestinal strongylosis control. We have evaluated the potential of three predatory mite species—*Macrocheles robustulus*, *Macrocheles muscaedomesticae* and *Rhabdocarpais consanguineus*—to reduce transmission of the nematode *Haemonchus contortus* by preying on infective larvae.

**Methods:**

Under laboratory conditions, mites were exposed to mixed prey environments containing housefly *Musca domestica* eggs and *H. contortus* third-stage (L3) larvae. Predation was first monitored over 8 h. This was followed by a more detailed assessment of *M. robustulus* predatory capacity in sheep feces containing eggs of *H. contortus* and then the oviposition capacity of females was studied when fed exclusively with L3 larvae.

**Results:**

*Macrocheles robustulus* demonstrated a significant preference for nematode larvae, leading to further evaluation of its predatory capacity in sheep feces containing 2250 eggs per gram of *H. contortus*. After 14 days at 25 ± 2 °C, the presence of *M. robustulus* resulted in a significant reduction of L3 larvae compared to controls (519 vs. 1067 L3 larvae; *p* < 0.05). Additionally, *M. robustulus* females oviposited when fed exclusively on nematode larvae.

**Conclusions:**

These findings suggest that *M. robustulus* could be a viable biological control agent against *H. contortus* in pasture settings, warranting further field studies.

**Graphical abstract:**

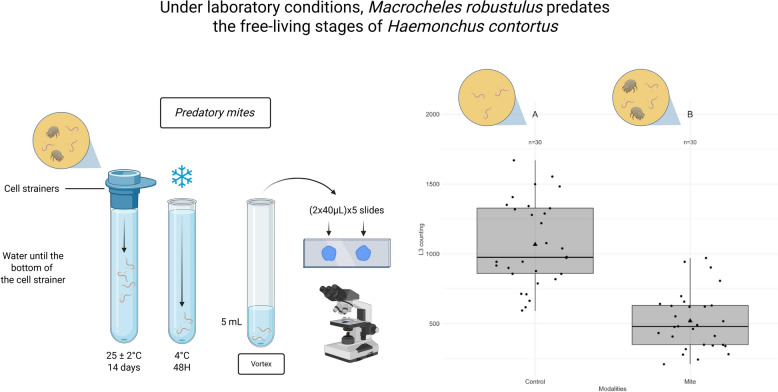

## Background

In small ruminant farming, the systematic use of anthelmintics faces increasing challenges, including growing consumer distrust of these treatments and the emergence of resistance [1–3]. This issue is particularly concerning for gastrointestinal strongyles (GIS), such as the nematode *Haemonchus contortus* [4–7]. Additionally, sheep farmers are becoming more aware of the negative environmental impact of these molecules on surrounding ecosystems due to the release residues and the effect on nontargeted fauna. In this context, adapting treatment recommendations for small ruminants and developing alternative parasite control methods have become urgent priorities. To address this challenge, various integrated parasite control strategies have been explored, including genetic selection for parasite-resistant and/or resilient sheep [8], targeted selective treatments using effective anthelmintics [9, 10] and pasture management strategies such as mixed grazing with cattle and small ruminants [11, 12]. When resistance decreases the effectiveness of conventional treatments, these approaches provide valuable tools to reduce reliance on chemotherapeutic solutions. However, despite their effectiveness, these methods alone may not be sufficient, particularly under high infection pressures.

Biological control has emerged as a promising alternative to chemical treatments [13]. This method was initiated primarily to control pests which were resistant to insecticides, and it relies on beneficial organisms to suppress pest populations. Biological control strategies have been widely implemented for protecting crops since 1970, initially in greenhouses and now increasingly in open-field agriculture systems [14]. Biological control agents are selected not only for their effectiveness but also for their precision in targeting pests, reducing impacts on non-target species and contributing to biodiversity conservation. In the livestock industry, the use of biological control strategies is also progressing, particularly for the management of flies, red mites and darkling beetles, contributing to a reduction in the use of chemical treatments [15, 16]. Among the beneficial organisms used in biological control, predatory mites are of particular interest due to their ability to effectively suppress pest populations and are currently widely used in agriculture to control a range of plant pests [17, 18]. It is also possible that several commercially available predatory mite species may play a role in the control of nematode populations, as suggested by Rueda-Ramírez et al. [19]. For this reason, the use of predatory mites against pathogenic nematodes could be of interest. This approach has recently emerged and has been studied by two different teams in France and Brazil [20, 21]. In addition to their established role in crop protection, certain predatory mite species also prey on nematodes, positioning them as potential candidates for the control of *H. contortus* in pastures. Investigating the feasibility of introducing predatory mites into pasture-based systems could open new avenues for integrated parasite control, reducing dependence on chemical treatments while enhancing sustainable livestock production.

To assess the potential of commercially available predatory mites as biological control agents against the free-living stages of *H. contortus*, we selected three mite species from the order Mesostigmata for study: *Macrocheles muscaedomesticae*, *Macrocheles robustulus* and *Rhabdocarpais consanguineus*. The objective was to evaluate their ability to prey on *H. contortus* third-stage (L3) infective larvae and determine their suitability for biocontrol applications. *Macrocheles muscaedomesticae* has been studied as a biocontrol agent since 1964 when Filipponi [22] first discussed its potential for fly control, subsequently demonstrated by Abo-Taka et al. [23]. *Macrocheles robustulus* (Berlese) has been commercially available since 2010 for the control of thrips and soil-dwelling fly larvae such as those of the family Sciaridae [17, 24, 25]. Both species are closely associated with coprophagous insects. *Macrocheles muscaedomesticae* is phoretic on dipteran pests such as the housefly *Musca domestica*, consuming its eggs and early larvae [26, 27], but it is also found on dung beetles [28, 29]. In comparison, *M. robustulus* is primarily phoretic on dung beetles [30] and is a generalist predator [31]. Both mite species are characterized by short generation times and both arrhenotokous parthenogenetic reproduction and sexual reproduction [32–35]. Their life-cycle consists of five stages: egg, larva, protonymph, deutonymph and adult [36]. *Rhabdocarpais consanguineus* was originally described as *Parasitus consanguineus* (Oudemans & Voigts, 1904) and later reclassified [37]. It has been identified in mushroom cultivation environments, where it preys on small organisms [38]. It can complete its development when fed on larvae of the mushroom sciarid flies *Megaselia halterata* and *Lycoriella ingenua*, suggesting its potential as a biological control agent in mushroom production [39]. However, little is known about its ability to prey on nematodes. Its congener *Parasitus bituberosus* has been shown to prey on various microorganisms, including rhabditid nematodes, which may indicate a similar capability in *R. consanguineus* [38, 40]. Unlike *Macrocheles* species, *Parasitus* species require fertilization for reproduction due to their diploid-diploid sex determination system [41].

These three mite species, used in biological control methods, are polyphagous predators and potential candidates for controlling *H. contortus*. Thus the aim of this study was to evaluate their ability to target *H. contortus* free-living stages under laboratory conditions. The key criteria assessed included attraction to *H. contortus* larvae despite alternative food sources, predation efficiency in feces and reproductive viability on a nematode-only diet.

To investigate these traits, we conducted a host–prey preference test on agar plates, comparing the prey choices of each predator between *H. contortus* L3 larvae and *M. domestica* eggs. Predatory capacity was further evaluated by measuring their ability to consume *H. contortus* larvae in sheep feces. Finally, we assessed their oviposition potential when fed exclusively on *H. contortus* L3 larvae. The goal was to identify a commercially available soil-dwelling predatory mite capable of effectively controlling *H. contortus* in pastures and explore its potential as a sustainable biocontrol agent in sheep farming.

## Methods

### Laboratory stock of predatory mites and preliminary experimental conditions

Females of three mite species—*M. muscaedomesticae*, *M. robustulus*, and *R. consanguineus*—were obtained from a commercial mass-rearing facility (Bestico, Pont-Saint-Martin, France). They were maintained in Petri dishes filled with a mixture of vermiculite and potting soil. Each colony was kept under dark conditions at 8 °C in a climatic chamber to minimize activity while ensuring survival. Colonies were provided with *M. domestica* eggs as food and moistened every 2 weeks.

One week before each experiment, mites from each species were transferred to 3.5-cm-diameter Petri dishes containing a 2% aqueous agar layer and placed in an incubator at 25 ± 2 °C in the dark. The agar was kept moist by the daily addition of distilled water. The open ends of the dishes were sealed with transparent plastic lids to prevent mite escape. To avoid conditioning mites to exclusively feed on fly eggs, they were provided with 40 μl of water daily containing a high density of L3 larvae for 5 days, followed by a 2-day starvation period before the experiment.

### Collection of infected feces and sample processing

Fresh feces containing *H. contortus* eggs were collected from artificially infected sheep housed indoors at the experimental facilities of the Ecole Nationale Vétérinaire de Toulouse (ENVT). Samples were obtained directly from each sheep’s rectum. Fecal egg counts (FEC) were performed using the modified McMaster technique, with 3 g of feces diluted in 42 ml of a saturated NaCl solution (density 1.2). The diagnostic sensitivity of this method is 15 eggs per gram of feces (EPG) [42].

### Source of* H. contortus* L3

Approximately 100 g of fecal sample from each *H. contortus*-infected sheep was incubated for at least 12 days at 25 ± 2 °C, with humidity maintained by the addition of tap water every 2–3 days. For larval collection, fecal samples were placed in containers filled to the brim with tap water, then inverted onto Petri dishes also filled with tap water [43]. This set-up was left at room temperature for 48 h, following which larvae were harvested. The collected larvae were stored at 4 °C for 48 h before the supernatant was removed by pipetting, leaving a 5-ml pellet containing the larvae. After vortexing, larvae in each pellet were counted by examining ten 40-μl aliquots of the pellet under a microscope. The larval concentration was calculated as the average of eight aliquots, excluding the highest and lowest values. The suspension was then diluted with tap water to prepare doses of approximately 240 L3 larvae in 40 μl of water.

### Host–prey preferences of predatory mites

The host–prey preferences of predatory mites were studied using agar plates prepared as described in section Source of* H. contortus* L3, in an incubator maintained at 25 ± 2 °C. For each mite species, a single mite was placed in the simultaneous presence of four *M. domestica* eggs and five *H. contortus* L3 larvae. Each Petri dish was considered to be an experimental unit, with 20 replicates per predatory mite species. The units were examined under a binocular microscope at 2-h intervals over 8 consecutive hours (t2h, t4h, t6h, t8h).

Prey consumption was determined by counting the number of missing *H. contortus* larvae and *M. domestica* eggs. The fly eggs were considered to be consumed if they were absent or showed visible signs of predation. At each observation, any consumed prey was replaced, regardless of type. The number of mites that had consumed at least one prey item was also recorded.

### Evaluation of* M. robustulus* predation on free-living* H. contortus* larvae in feces

To evaluate the predation effect of *M. robustulus* on free-living *H. contortus* larvae in feces, we prepared and processed the experimental units following the methodology of Grisez et al. [20] with minor modifications. A total of 60 experimental units were set up, each containing 1 g of feces containing a *H. contortus* egg concentration of 2250 EPG from the same coprological sample. One-half of the units were colonized with five mites each, while the other half remained mite-free (control units).

After 14 days in an incubator at 25 ± 2 °C under dark and humid conditions, the number of unhatched *H. contortus* eggs within the feces and the number of L3 larvae that had migrated were counted for each unit, following the protocol of Grisez et al. [20].

### Observation of oviposition by * M. robustulus* females fed a diet of* H. contortus* L3 larvae

A single *M. robustulus* female was placed on an agar Petri dish, as previously described, with dry sphagnum moss serving as shelter and oviposition sites. The experiment was conducted under dark conditions in an incubator maintained at 25 ± 2 °C. Each Petri dish containing a mite was considered to be an experimental unit. Ten replicates were provided, with a daily application of approximately 240 *H. contortus* L3 larvae in 40 µl of water; ten control replicates received only 40 µl of water per day without L3 larvae. All units were examined daily for 2 weeks under a stereomicroscope to detect and count eggs or larvae.

### Statistical analysis

The statistical analyses were performed using R. 4.3.0 and RStudio (R Foundation for Statistical Computing, Vienna, Austria). *P*-values < 0.05 were considered to indicate statistical significance. To characterize diet according of each mite species, we performed Fisher’s exact tests on contingency tables comparing at each time point (t2h, t4h, t6h, t8h) a theoretical population having no prey preference with our observed populations. As each sample was composed of 20 individuals per species, our theoretical population had 10 individuals for each modality. Experiments were based on the null hypothesis (H0) that there was no significant difference between the theoretical population and observed populations. In addition, bar plots were created to show the number of individuals by condition for each species at each time point. For each observation, the percentage of mites that predated at least one prey was recorded.

For the assessment of *M. robustulus* predatory capacity on *H. contortus* free-living stages, we compared larval counts between mite and non-mite experimental units. We created a generalized linear model (GLM) using Poisson distribution, following which then, analysis of variance (ANOVA) (Tukey’s test) was used to compare the number of recovered nematode larvae between the units with mites and the control units.

After the nematode eggs remaining in the filter had been counted, the average hatching percentage of *H. contortus* eggs contained in 1 g of feces from the 30 experimental units was calculated for both modalities. Box plots were also created to show the numbers of larvae that migrated through the filter for each modality. For assessment of mite oviposition, binomial sampling based on the presence (1) or absence (0) of juveniles was investigated for estimating the reproductive capacity of *M. robustulus*, followed by a proportion test to compare the number of juveniles between mite control units and the mite units fed L3 larvae.

## Results

### Host–prey preferences of predatory mites

**Fig. 1:**
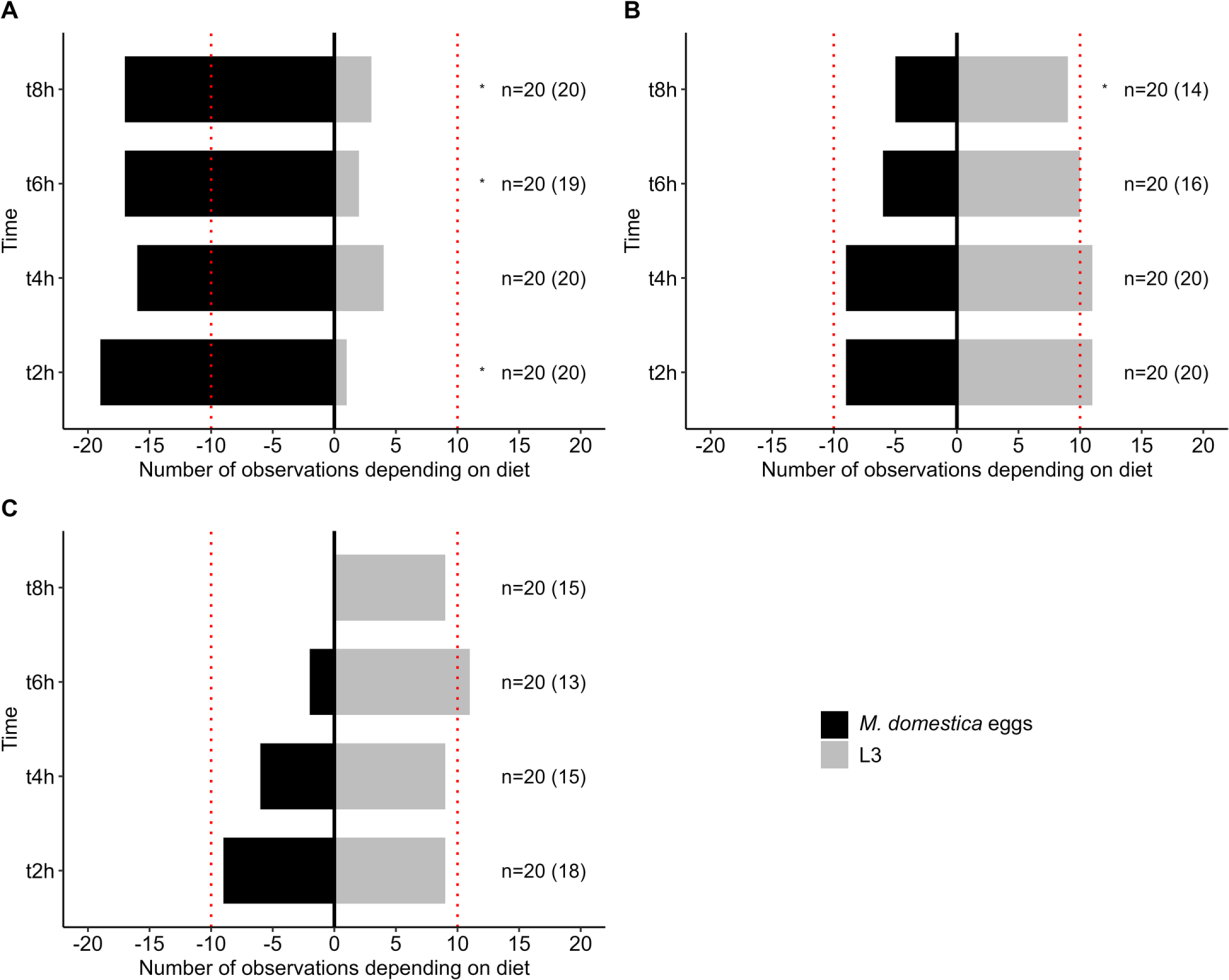
Host–prey preferences of predatory mites. Prey preference of the predatory mites *Macrocheles muscaedomesticae* (**A**), *Macrocheles robustulus* (**B**) and *Rhabdocarpais consanguineus* (**C**) when offered a choice between the housefly *Musca domestica* eggs and the nematode *Haemonchus contortus* L3 larvae. The asterisk (*) indicates a statistically significant difference at *p* < 0.05 between the observed diet of mites and the expected diet of a neutral (non-preferential) population. Numbers in parentheses represent the number of mites that consumed at least one prey item out of the total sample (*n* = 20). L3, Third-stage larvae

The analysis of prey selection revealed a clear preference of *M. muscaedomesticae* for fly eggs at all observation times (Fig. 1A). Fisher’s exact tests confirmed this trend, with significant differences at t2h (odds ratio = 0.05665192, IC 95% [0.001159847; 0.495179813], p = 0,003342), t6h (odds ratio = 0.1246367, IC 95% [0.01110633; 0.75480267], p = 0.01381) and t8h (odds ratio = 0.184812 , IC 95% [0.02632766; 0.94457354], p = 0.04074) compared to an unbiased prey preference. At t4h, the *p*-value was 0.09585 (odds ratio = 0.2593023 , IC95% [0.04602295; 1.20945268]), approaching significance. Additionally, 95–100% of *M. muscaedomesticae* individuals fed, regardless of the observation period.

*Macrocheles robustulus* exhibited a more balanced prey selection than *M. muscaedomesticae*, showing no significant prey preference at t2h, t4h, and t6h (Fig. 1B). However, at t8h, *M. robustulus* displayed a significant preference for *H. contortus* L3 larvae ((odds ratio = 0.184812 , IC 95% [ 0.02632766 0.94457354 ], p = 0.04074) ). Feeding rates for *M. robustulus* were 100% at t2h and t4h, decreasing to 80% at t6h and 70% at t8h.

The preference of *Rhabdocarpais consanguineus* remained balanced across all observation times (Fig. 1C). Fisher’s exact tests confirmed this, with *p*-values > 0.05 indicating no significant prey preference (t2h (odds ratio =1, IC 95% [0.2336435;4.2800245], p = 1), t4h (odds ratio = 1.482632, IC 95% [0.3197625; 7.2399004], p = 0.7338), t6h (odds ratio = 5.224665 , IC 95% [0.8126125; 60.5750327], p = 0.06715), t8h (odds ratio = 1.482632 , IC 95% [0.3197625;7.2399004], p = 0.7338)) . The percentage of *R. consanguineus* individuals that fed was 90% at t2h, 75% at t4h, 65% at t6h, and 75% at t8h.

The prey selection experiment identified *M. robustulus* as a distinct predator, leading to its selection for further evaluation of its predatory capacity against free-living *H. contortus* larvae (L3) in feces.

### Evaluation of* M. robustulus* predation on free-living* H. contortus* larvae in feces

In this test we evaluated the predation effect of *M. robustulus* on free-living *H. contortus* larvae (L3) in feces. The presence of *M. robustulus* did not affect the hatching success of *H. contortus* eggs, as nearly all eggs hatched into larvae in both the control and mite-exposed conditions (99.36% and 99.05%, respectively). This confirms that *H. contortus* eggs successfully developed into larvae despite the presence of the predatory mite *M. robustulus*.

**Fig. 2:**
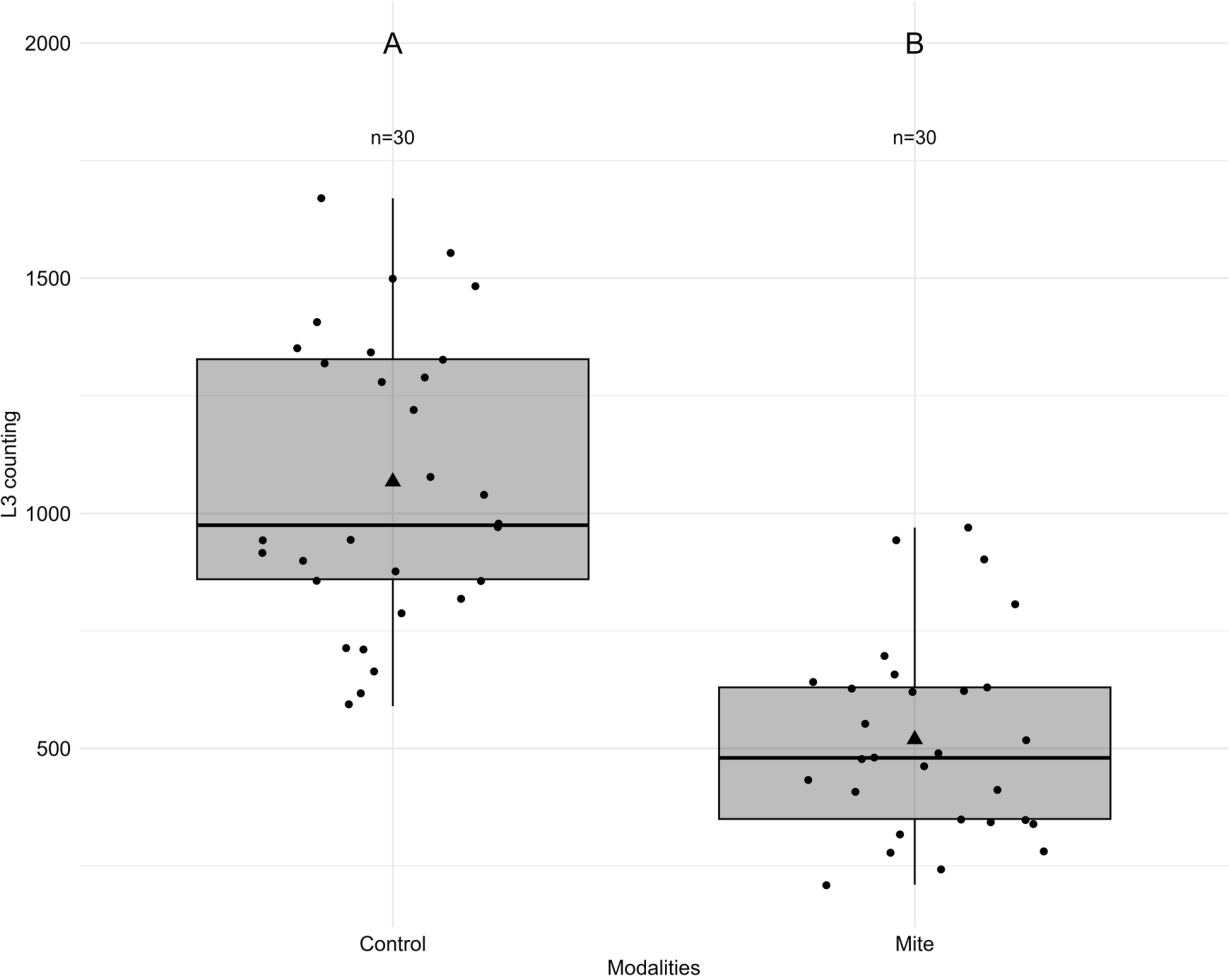
Impact of *Macrocheles robustulus* on the survival of *Haemonchus contortus* L3 larvae. Survival rate of *H. contortus* L3 larvae in experimental units with *M. robustulus* compared to control units without mites. Boxes represent the interquartile range, with the median (horizontal line) and mean (triangle). Vertical lines indicate the range of variation, excluding outliers (dots). Different uppercase letters (e.g. **A**, **B**) indicate significant differences (*p* < 0.05; Tukey’s test) between groups. L3, Third-stage larvae

However, the number of L3 larvae collected was significantly lower in the experimental units with *M. robustulus* compared to the control units. In the control units (without mites), an average of 1067 L3 larvae were recovered, compared to only 519 L3 larvae in the mite-exposed units (Fig. 2). This difference was statistically significant (F(1,57) = 66.797, p <0.001)   suggesting that *M. robustulus* actively preyed upon larvae, reducing their numbers. While *M. robustulus* did not interfere with the hatching of *H. contortus* egg, the presence of this mite significantly reduced the number of L3 larvae in sheep feces, indicating its potential as a biological control agent against free-living *H. contortus* larvae in feces.

### Observation of* M. robustulus* oviposition on a diet of* H. contortus* L3 larvae

When provided with *H. contortus* L3 larvae as a food source, *M. robustulus* demonstrated the ability to reproduce. One juvenile mite was observed in six out of 10 experimental units where mites were fed with larvae, whereas no offspring were detected in the control group deprived of L3 larvae. These results indicate that *H. contortus* constitutes a suitable diet for *M. robustulus.*

## Discussion

The aim of this study was to adopt a pragmatic approach by focusing on commercially available beneficial arthropods. Given the potential of acarofauna present in pastures, the selection of candidate species was guided by their availability on the market, leading to the choice of *M. muscaedomesticae*,* M. robustulus* and *R. consanguineus*. In this context, and with the purpose of identifying a predatory mite species suitable for the biological control of *H. contortus*, we compared the predatory potential of *M. muscaedomesticae*, *M. robustulus* and *R. consanguineus* on L3 larvae of the nematode *H. contortus*. Among the three species assessed for prey preference, *M. robustulus* and *R. consanguineus* exhibited higher feeding rates on L3 larvae than *M. muscaedomesticae*.

In our trials, *M. muscaedomesticae* exhibited a clear preference for fly eggs over *H. contortus* L3 larvae. This tendency to prioritize eggs as a food source aligns with findings from Azevedo et al. [31], who reported that while *M. muscaedomesticae* consumed various prey types, its predation rates were highest on eggs of *Stomoxys calcitrans* and *M. domestica* eggs, rather than on the first instar larvae of these dipteran flies.

Unlike *M. muscaedomesticae*, *R. consanguineus* exhibited no strong prey preference across the observation periods, suggesting a more generalist feeding strategy. While this trait appeared promising for biological control, its predation activity remained inconsistent, as individuals frequently remained immobile or moved along the lid of the Petri dish rather than on the agar where prey were located. Given that predation relies on chance encounters [44, 45], this behavior reduced its potential for effective predation on L3 larvae or fly eggs. Consequently, it was difficult to determine whether the absence of predation resulted from satiation, a foraging failure or disruptive conditions. Due to these uncertainties, we discontinued our investigation with this species.

*Macrocheles robustulus* also exhibited a balanced diet throughout most of the observation periods, with the exception of the final time point (t8h), where it displayed a significant preference for L3 larvae. While 35% of individuals did not engage in predation, this inactivity may have been due to satiation rather than behavioral constraints. In contrast to *R. consanguineus*, *M. robustulus* actively moved across the agar surface, frequently encountering L3 larvae and fly eggs but selectively ignoring the latter. This shift in prey selection at t8h suggests that when satiated, *M. robustulus* may opt for smaller, easier-to-consume prey, such as L3 larvae.

The polyphagous nature of *M. robustulus* suggests a greater capacity for survival in pasture ecosystems, where prey availability fluctuates. By preying on both *H. contortus* L3 larvae and fly eggs—two organisms commonly found in feces—this species may contribute to the suppression of *H. contortus* populations while maintaining its own sustenance in the absence of sufficient L3 larvae. This ecological flexibility aligns with findings from similar studies, which have shown that dietary diversity can enhance the abundance of predatory mites [46]. Moreover, food supplementation has been demonstrated to improve the biological control efficiency of *Neoseiulus cucumeris* in both laboratory and greenhouse experiments [47].

Once *M. robustulus* was identified as a promising candidate, we evaluated its predatory capacity against free-living *H. contortus* larvae directly in feces. Since all fecal samples originated from the same coprological batch and experimental units were incubated under identical conditions, the significant reduction in L3 larvae in mite-exposed units can be attributed to *M. robustulus* predation on the free-living stages of the nematode.

Our findings contradict those of Araújo dos Anjos et al. [21], who did not observe predation by *M. robustulus* on *H. contortus* larvae in feces. In their study, feces and vermiculite were mixed and saturated with 10 ml of distilled water, but vermiculite was only sparsely added to *M. robustulus* experimental units, whereas other mite species were provided with a more aerated substrate [48]. This difference in the proportion of vermiculite may have altered the structure of the medium, possibly restricting the access of *M. robustulus* to prey, as these mites rely on substrate porosity to efficiently locate and capture their prey within feces [49]. In natural pastures, fecal matter is aerated by the activity of coprophagous insects such as those belonging to orders Diptera and Coleoptera [50, 51], facilitating mite predation. In addition to the possible locomotion difficulties of the mite, the efficacy of *M. robustulus* as a biocontrol agent may also depend on the origin of the population. Commercial strains, often selected for mass rearing, could exhibit different predatory behaviors compared to wild populations [25]. For example, the Brazilian population studied by Dos Anjos et al. [21] showed no predation on *H. contortus* larvae, possibly due to substrate differences or local adaptation to alternative prey. Such intraspecific variation is well-documented in biocontrol systems; for example, entomopathogenic fungi like *Beauveria bassiana* show strain-specific virulence [52]. Further studies comparing commercial and wild *M. robustulus* strains under standardized conditions would clarify these potential differences and optimize field applications.

In addition to assessing predation, we evaluated whether a diet composed exclusively of *H. contortus* L3 larvae could support *M. robustulus* reproduction. The reproductive success of mites is strongly influenced by external factors such as temperature, humidity and nutrient availability [53]. To optimize conditions, we provided a Petri dish set-up with dry sphagnum moss as oviposition shelters and agar to maintain prey viability, both of which are essential factors for successful reproduction [36]. Under these conditions, *M. robustulus* oviposition was observed in six out of 10 units, confirming that *H. contortus* L3 larvae alone can sustain reproduction.

However, the presence of sphagnum moss, while beneficial for oviposition, prevented direct egg counts due to their small size (approximately 0.3 × 0.2 mm) [32]. Consequently, only juvenile mites were recorded. The relatively low number of juveniles observed may indicate egg mortality or suboptimal laboratory conditions, particularly in the absence of dietary supplementation. In natural pasture systems, the availability of organic matter and diverse prey sources within dung is likely to provide a more balanced nutrient intake, supporting higher reproductive success [19].

## Conclusion

The exploration of dung beetle-associated predatory mites as natural enemies of free-living *Haemonchus contortus* stages [20] has opened new prospects for integrating predatory mites into gastrointestinal nematode (GIN) management strategies. Successful biological control requires releasing a sufficient number of natural predators to effectively reduce the target population in a specific area [54]. Our study highlights *Macrocheles robustulus* as a promising biocontrol agent for managing GINs in sheep farming, particularly because it is already commercially mass-produced. To validate its practical application, further investigations under semi-field and field conditions are essential to assess its ability to establish and reproduce in situ while evaluating potential impacts on native dung beetle and mite populations.

## Data Availability

Data supporting the main conclusions of this study are included in the manuscript.

## Abbreviations

EPG: Eggs per gram of feces
FEC: Fecal egg counts
GIN: Gastrointestinal nematode
L3 larvae: Free-living third-stage larvae
